# Modifiable Risk Factors for Symptom Burden in Chronic Rhinosinusitis With Nasal Polyps: The Role of Obesity and Sleep Apnoea

**DOI:** 10.1111/coa.70058

**Published:** 2025-11-05

**Authors:** Javier Modesto García‐Fernández, María Soledad Sánchez‐Torices, María Pilar Gómez‐Gallego, Miguel Ángel Feliz‐Fernández, María Alharilla Montilla‐Ibáñez, Rafael Lomas‐Vega

**Affiliations:** ^1^ Department of Health Sciences University of Jaén Jaén Spain; ^2^ Department of Otolaryngology Hospital Universitario de Jaén Jaén Spain

**Keywords:** body mass index, nasal polyps, quality of life, rhinosinusitis, sleep apnoea, systemic inflammation

## Abstract

**Objective:**

To identify sociodemographic and clinical predictors of symptom severity and quality of life (QoL) impairment in patients with chronic rhinosinusitis with nasal polyps (CRSwNP), diagnosed according to the latest EPOS 2020 criteria.

**Study Design:**

Cross‐sectional analytical study.

**Setting:**

Tertiary Care Center (University Hospital of Jaén, Spain).

**Methods:**

A total of 188 patients diagnosed with CRSwNP were evaluated. Symptom severity and QoL were assessed using the Nasal Obstruction Symptom Evaluation (NOSE‐E) and the Sino‐Nasal Outcome Test (SNOT‐22). Multiple linear regression analysis was used to identify predictors of symptom burden.

**Results:**

Higher body mass index (BMI) and the presence of sleep apnoea were independently associated with increased symptom severity, as measured by both the NOSE‐E (adj. *R* ^2^ = 0.224, *p* = 0.002) and the SNOT‐22 (adj. *R* ^2^ = 0.242, *p* = 0.005). Smoking was associated in the bivariate analysis but was not retained in the multivariate models.

**Conclusion:**

BMI and sleep apnoea are independent predictors of greater symptom burden and poorer QoL in CRSwNP. These findings highlight the importance of incorporating weight management and sleep apnoea screening into the multidisciplinary management of CRSwNP to improve patient outcomes.

**Summary**

- BMI and sleep apnoea as key predictors
  ○ Higher body mass index (BMI) and the presence of obstructive sleep apnoea (OSA) were independently associated with increased symptom burden and poorer quality of life in patients with chronic rhinosinusitis with nasal polyps (CRSwNP), as measured by NOSE‐E and SNOT‐22 scores.
- Limited role of smoking
  ○ Although smoking showed significance in univariate analyses, it did not retain predictive value in multivariate models, suggesting a weaker independent role compared to obesity and OSA.
- Symptom domain analysis
  ○ BMI significantly impacted all five SNOT‐22 symptom domains, while OSA was selectively associated with nasal, otologic/facial and functional/emotional domains, but not with sleep dysfunction or emotional quality of life.
- Clinical implications
  ○ The findings support incorporating weight management and OSA screening into CRSwNP treatment strategies, emphasising a multidisciplinary approach to improve patient outcomes.
- Study strengths and limitations
  ○ The use of validated PROMs in a real‐world clinical population enhances generalisability, but the cross‐sectional design limits causal inference, and objective inflammatory or anatomical markers were not assessed.

## 1 Introduction

Chronic rhinosinusitis with nasal polyps (CRSwNP) is a chronic inflammatory condition of the nasal and paranasal sinus mucosa, affecting approximately 2%–4% of the global population. The presence of persistent nasal polyps leads to nasal obstruction, rhinorrhoea, hyposmia and facial pressure, often resulting in a significant impairment of patients' quality of life (QoL). In addition to the personal health burden, CRSwNP imposes a notable economic impact due to its chronic nature, frequent need for corticosteroid therapy and surgical interventions such as endoscopic sinus surgery (ESS) [1, 2].

The assessment of symptom burden and disease severity in CRSwNP has been standardised using validated patient‐reported outcome measures (PROMs), notably the Sino‐Nasal Outcome Test (SNOT‐22) and the Nasal Obstruction Symptom Evaluation (NOSE) scale [3, 4]. These tools provide quantifiable and reproducible data on sinonasal symptoms, their functional impact and overall QoL.

From a pathophysiological standpoint, CRSwNP is predominantly associated with Type 2 inflammation, characterised by overexpression of interleukins IL‐4, IL‐5 and IL‐13, leading to eosinophilic infiltration and tissue remodelling [5]. Comorbidities such as asthma, allergic rhinitis and nonsteroidal anti‐inflammatory drug (NSAID) intolerance are frequent and often correlate with a more severe disease phenotype [6]. Moreover, systemic conditions like obesity and obstructive sleep apnoea (OSA) have been increasingly associated with upper airway inflammation and poorer sinonasal outcomes [7].

Despite the growing understanding of inflammatory mechanisms and the role of biologic treatments, less is known about the clinical and demographic factors that independently predict symptom severity and QoL impairment in CRSwNP. In particular, the interaction between modifiable factors such as body mass index (BMI), sleep apnoea and lifestyle habits (e.g., smoking) remains underexplored in large clinical samples [8].

Previous studies have identified associations between obesity and sinonasal inflammation [8, 9], as well as between OSA and chronic rhinosinusitis severity [7, 10]. However, few investigations have assessed these factors simultaneously using validated outcome measures such as NOSE‐E and SNOT‐22.

In this context, the present study aims to identify clinical and sociodemographic predictors of symptom severity in patients with CRSwNP, with a focus on modifiable systemic factors such as BMI, sleep apnoea and smoking. We hypothesise that obesity and OSA are independently associated with more severe sinonasal symptoms and reduced QoL. By clarifying these relationships, we aim to support a more integrated, multidisciplinary approach to managing CRSwNP.

## 2 Material and Methods

### 2.1 Study Design and Setting

This cross‐sectional analytical study included 188 consecutive patients diagnosed with CRSwNP, recruited between May and December 2024 at the University Hospital de Jaén (Spain). The research protocol was approved by the Ethics committee of the Junta de Andalucía (Study Codec SICEIA‐2024‐003273) and was designed and conducted in accordance with the Ethics code of the World Medical Association for studies including human participants (Declaration of Helsinki). All participants were provided with written informed consent to obtain voluntary agreement to participate in the study.

### 2.2 Participants and Eligibility Criteria

A total of 188 consecutive adult patients diagnosed with chronic rhinosinusitis with nasal polyps (CRSwNP) were recruited from outpatient otolaryngology clinics. Diagnosis was established according to the criteria set by the European Position Paper on Rhinosinusitis and Nasal Polyps 2020 (EPOS 2020) [2]. Inclusion required the presence of at least two symptoms, one of which had to be either nasal obstruction or nasal discharge (anterior or postnasal), and could include facial pressure or pain, and/or reduction or loss of smell, lasting for at least 12 weeks. Diagnosis was confirmed by nasal endoscopy showing visible polyps in the middle meatus, and by computed tomography (CT) revealing mucosal changes in the paranasal sinuses and/or ostiomeatal complex. Objective severity measures such as endoscopic (Lund–Kennedy) or radiologic (Lund–Mackay) scores were not collected in this study, as the primary aim was to identify clinical and demographic predictors of patient‐reported symptom burden, rather than to evaluate anatomical disease severity. This methodological choice allowed us to focus on patient‐centred outcomes and modifiable systemic comorbidities.

All evaluations were conducted by board‐certified otolaryngologists specialised in rhinology to ensure diagnostic accuracy.

### 2.3 Sample Size Estimation

The sample size was determined based on recommendations for multiple linear regression analysis, requiring 10–20 subjects per independent variable [11]. With 12 predictors considered in the model, a minimum of 120 participants was necessary. A final sample of 188 subjects was deemed sufficient to ensure adequate statistical power.

### 2.4 Data Collection and Study Variables

Data were collected through structured clinical interviews and direct anthropometric measurements. Outcome measures were collected during scheduled outpatient visits. The study population was intentionally heterogeneous, including both newly diagnosed patients and individuals with a longer disease course. Some participants had undergone prior ESS, were on intranasal or systemic corticosteroids, or were receiving biologic therapy. This diversity in treatment status reflects the real‐world clinical spectrum of CRSwNP and allows for the identification of predictors across a broad range of patient profiles. The following variables were recorded:

- *Sociodemographic variables*: age (in years), gender (male/female) and educational level (primary, secondary, university).
- *Lifestyle variables*: current smoking status (yes/no), passive exposure to tobacco (living with smokers: yes/no) and pet ownership at home (yes/no).
- *Clinical history*: presence of asthma or allergy (confirmed by prior medical diagnosis), OSA confirmed by overnight polysomnography and documented in the medical record (yes/no), history of previous nasal surgery (yes/no) and surgical indication for ESS based on clinical evaluation (yes/no).
- *Anthropometrics*: weight (kg) and height (cm) were measured using calibrated devices (Tefal digital scale and T201‐T4 adult stadiometer, respectively). BMI was calculated as weight in kilograms divided by height in metres squared (kg/m^2^) and classified as follows: normal (< 25 kg/m^2^), overweight (25–29.9 kg/m^2^) and obese (≥ 30 kg/m^2^) according to WHO rules.

### 2.5 Outcome Measures

Symptom burden and QoL were assessed using two validated PROMs:

- *NOSE‐E—Spanish version*: This scale assesses the severity of nasal obstruction and its impact on daily functioning. It includes 10 items scored on a 5‐point Likert scale (0 = *no problem* to 4 = *severe problem*), with a total score ranging from 0 to 40. The scale has shown excellent internal consistency (Cronbach's *α* = 0.86–0.90) and strong construct validity in previous studies [12]. Patients completed the questionnaire independently in a quiet setting, with investigators available for clarification if needed.
- *SNOT‐22*: This instrument evaluates a broad range of symptoms including nasal, extranasal, sleep‐related and emotional domains. Each of its 22 items is scored from 0 to 5, yielding a maximum total score of 110. Higher scores indicate greater symptom burden and worse QoL. The scale has demonstrated high internal consistency (Cronbach's *α* = 0.89–0.91) and responsiveness to change [13].

### 2.6 Statistical Analysis

Data were analysed using SPSS version 27 (IBM Corp., Armonk, NY, USA). Continuous variables were expressed as means and standard deviations (SD), and categorical variables as frequencies and percentages. The Kolmogorov–Smirnov test was used to assess the normality of continuous data.

Bivariate associations between predictor variables and the two outcome measures (NOSE‐E and SNOT‐22 scores) were examined using univariate linear regression. Variables with *p* < 0.10 in bivariate analyses were entered into stepwise multiple linear regression models. In the final multivariate model, variables were retained if *p* < 0.05.

Model assumptions (linearity, normality of residuals, homoscedasticity) were evaluated using graphical methods and statistical diagnostics. Multicollinearity was assessed through condition indices and variance inflation factors (VIF). Effect size was interpreted using the adjusted coefficient of determination (adjusted *R* ^2^), and according to Cohen's criteria: < 0.02 (negligible), 0.02–0.15 (small), 0.15–0.35 (moderate) and > 0.35 (large).

### 2.7 Sub‐Analysis by Symptom Domains of the SNOT‐22

Domain‐specific analyses were exploratory and post hoc, designed to characterise symptom profiles and to examine the specific impact of BMI and OSA on SNOT‐22 domains. Based on established domain structures described in previous literature (e.g., Hopkins et al.), the 22 items of the SNOT‐22 were grouped into five domains: nasal symptoms, otologic/facial pain, sleep dysfunction, functional/emotional limitations and emotional QoL. For each patient, domain‐specific scores were calculated by summing the responses to items corresponding to each domain.

Linear regression models were constructed for each domain score as the dependent variable, with BMI (continuous) and presence of OSA (binary) as independent variables. Analyses were restricted to patients diagnosed with chronic rhinosinusitis with nasal polyps (CRSwNP). Model fit was assessed using adjusted *R* ^2^, and statistical significance was set at *p* < 0.05.

## 3 Results

**FIGURE 1:**
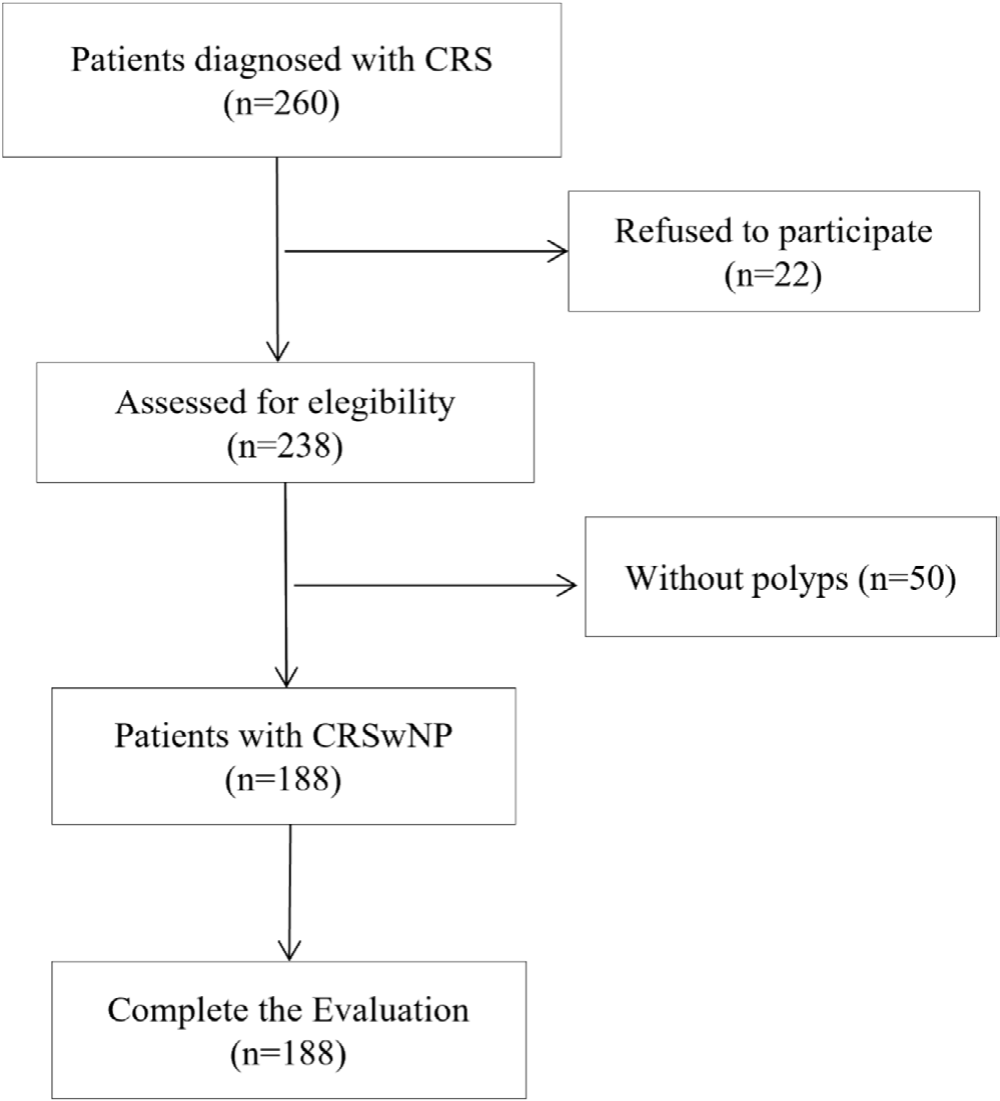
Flowchart illustrating patient selection and study inclusion.

The flow chart shows the process of subject selection and retention (Figure 1). A sample size of 188 subjects diagnosed with CRSwNP was recruited and responded to all questions and questionnaires. The sample's sociodemographic and clinical characteristics are detailed in Table 1. The mean age was 47 years (SD = 15), with 60.1% male participants. The average BMI was 26.4 kg/m^2^ (SD = 4.0), and the mean scores for the NOSE‐E and SNOT‐22 scales were 9 (SD = 4) and 39.7 (SD = 18.0), respectively.

**TABLE 1:** Sociodemographic and clinical characteristics of the study sample.

		Mean	SD	Frequencies	%
Age		47	15		
Weight		76	13		
Height		169	10		
Body mass index		26.44	3.99		
NOSE_E		9	4		
SNOT_22		39.66	17.99		
Gender	Male			113	60.1%
	Female			75	39.9%
Study level	Elementary Education			90	47.9%
	High School			59	31.4%
	University Education			39	20.7%
Smoker	Yes			53	28.2%
	No			135	71.8%
Living with smokers	Yes			48	25.5%
	No			140	74.5%
Sleep apnoeas	Yes			56	29.8%
	No			132	70.2%
Allergy	Yes			99	52.7%
	No			89	47.3%
Asthma	Yes			98	52.1%
	No			90	47.9%
Previous nasal surgeries	Yes			69	36.7%
	No			119	63.3%
Nasal surgery required	Yes			39	20.7%
	No			149	79.3%
Living with smokers	Yes			52	27.8%
	No			135	72.2%

### 3.1 NOSE‐E Score Predictors

In the univariate analysis, higher BMI (*β* = 0.438, *p* < 0.001), smoking (*β* = −0.148, *p* = 0.042) and presence of sleep apnoea (*β* = −0.296, *p* < 0.001) were significantly associated with higher NOSE‐E scores (Table 2). Age, gender, education level, comorbidities (allergy, asthma) and environmental exposures (pets, passive smoking) were not significantly related to NOSE‐E scores.

**TABLE 2:** Predictive variables for impact of symptoms measured with NOSE‐E. Multiple linear regression analysis predicting NOSE‐E scores.

NOSE‐E	B	Error	*β*	*t*	*p*	Li	Ls	B	Error	*β*	*t*	*p*	Li	Ls
Age	0.006	0.021	0.019	0.261	0.794	−0.036	0.047	—	—	—	—	—	—	—
Body mass index	0.475	0.072	0.438	6.647	< 0.001^a^	0.334	0.616	0.424	0.072	0.391	5.903	< 0.001^a^	0.282	0.566
Gender	−0.500	0.645	−0.057	−0.775	0.440	−1.772	0.773	—	—	—	—	—	—	—
Study level	0.530	0.402	0.096	1.318	0.189	−0.263	1.324	—	—	—	—	—	—	—
Smoker	−1.423	0.695	−0.148	−2.046	0.042^a^	−2.794	−0.051	NS	NS	NS	NS	NS	NS	NS
Living with smoker	0.920	0.722	0.093	1.274	0.204	−0.505	2.345	—	—	—	—	—	—	—
Living with animals	−0.578	0.708	−0.060	−0.816	0.416	−1.976	0.820	—	—	—	—	—	—	—
Sleep apnoeas	−2.797	0.661	−0.296	−4.233	< 0.001^a^	−4.100	−1.493	−1.951	0.624	−0.207	−3.125	0.002^a^	−3.182	−0.719
Allergy	−1.089	0.628	−0.126	−1.733	0.085	−2.329	0.151	—	—	—	—	—	—	—
Asthma	−0.371	0.633	−0.043	−0.587	0.558	−1.619	0.877	—	—	—	—	—	—	—
Previous nasal surgeries	−0.019	0.656	−0.002	−0.029	0.977	−1.314	1.275	—	—	—	—	—	—	—
Requires nasal surgery	−1.121	0.776	−0.105	−1.445	0.150	−2.651	0.409	—	—	—	—	—	—	—

*Note*: NOSE: Spanish version of the Nasal Obstruction Symptom Evaluation Scale.

Abbreviation: NS, no significance.

^a^ Statistical significance *p* < 0.05.

The multivariate regression model identified BMI (*β* = 0.391, *p* < 0.001) and sleep apnoea (*β* = −0.207, *p* = 0.002) as independent predictors of nasal obstruction severity. The model explained 22.4% of the variance in NOSE‐E scores (adjusted *R* ^2^ = 0.224, *p* = 0.002).

### 3.2 SNOT‐22 Score Predictors

Similarly, in the univariate analysis, BMI (*β* = 0.466, *p* < 0.001), smoking (*β* = −0.196, *p* = 0.007) and sleep apnoea (*β* = −0.283, *p* < 0.001) showed significant associations with SNOT‐22 scores (Table 3).

**TABLE 3:** Predictive variables for the impact of symptoms measured with SNOT‐22. Multiple linear regression analysis predicting SNOT‐22 scores.

SNOT‐22	*B*	Error	*β*	*t*	*p*	Li	Ls	*B*	Error	*β*	*t*	*p*	Li	Ls
Age	0.045	0.088	0.037	0.506	0.614	−0.130	0.219	—	—	—	—	—	—	—
Body mass index	2.101	0.293	0.466	7.177	< 0.001^a^	1.524	2.679	1.908	0.295	0.423	6.464	< 0.001	1.326	2.490
Gender	−2.850	2.678	−0.078	−1.064	0.289	−8.132	2.433	—	—	—	—	—	—	—
Study level	0.674	1.680	0.029	0.401	0.689	−2.640	3.988	—	—	—	—	—	—	—
Smoker	−7.805	2.867	−0.196	−2.723	0.007^a^	−13.460	−2.150	NS	NS	NS	NS	NS	NS	NS
Living with smoker	4.019	3.002	0.098	1.339	0.182	−1.903	9.941	—	—	—	—	—	—	—
Living with animals	−2.701	2.940	−0.067	−0.919	0.359	−8.502	3.099	—	—	—	—	—	—	—
Sleep apnoeas	−11.116	2.758	−0.283	−4.030	< 0.001^a^	−16.557	−5.675	−7.309	2.566	−0.186	−2.848	0.005	−12.372	−2.246
Allergy	−3.493	2.622	−0.097	−1.332	0.184	−8.665	1.679	—	—	—	—	—	—	—
Asthma	0.056	2.633	0.002	0.021	0.983	−5.138	5.250	—	—	—	—	—	—	—
Previous nasal surgeries	0.080	2.729	0.002	0.029	0.977	−5.303	5.464	—	—	—	—	—	—	—
Requires nasal surgery	−1.950	3.240	−0.044	−0.602	0.548	−8.343	4.443	—	—	—	—	—	—	—

*Note*: SNOT‐22: Spanish version of the Sino‐Nasal Outcome Test‐22.

Abbreviation: NS, no significance.

^a^ Statistical significance *p* < 0.05.

In the multivariate model, BMI (*β* = 0.423, *p* < 0.001) and sleep apnoea (*β* = −0.186, *p* = 0.005) remained significant independent predictors of worse QoL. The final model accounted for 24.2% of the variance in SNOT‐22 scores (adjusted *R* ^2^ = 0.242, *p* = 0.005).

Smoking lost statistical significance in both multivariate models, despite its association in univariate analysis.

### 3.3 Impact of BMI and Sleep Apnoea Across SNOT‐22 Symptom Domains

Subdomain analysis revealed differential effects of BMI and OSA across SNOT‐22 domains. Higher BMI was significantly associated with worse scores in all five domains, including nasal symptoms (*β* = 0.49, *p* < 0.001), otologic/facial symptoms (*β* = 0.26, *p* < 0.001), sleep dysfunction (*β* = 0.21, *p* = 0.001), functional/emotional limitations (*β* = 0.24, *p* = 0.0002) and emotional QoL (*β* = 0.65, *p* < 0.001).

The presence of OSA was also a significant predictor for nasal (*β* = 2.65, *p* = 0.0004), otologic (*β* = 1.43, *p* = 0.0015) and functional/emotional domains (*β* = 1.60, *p* = 0.0046), but not for sleep dysfunction (*p* = 0.64) or emotional QoL (*p* = 0.41). The adjusted *R* ^2^ values ranged from 0.05 (sleep) to 0.23 (nasal), indicating small‐to‐moderate effect sizes.

**FIGURE 2:**
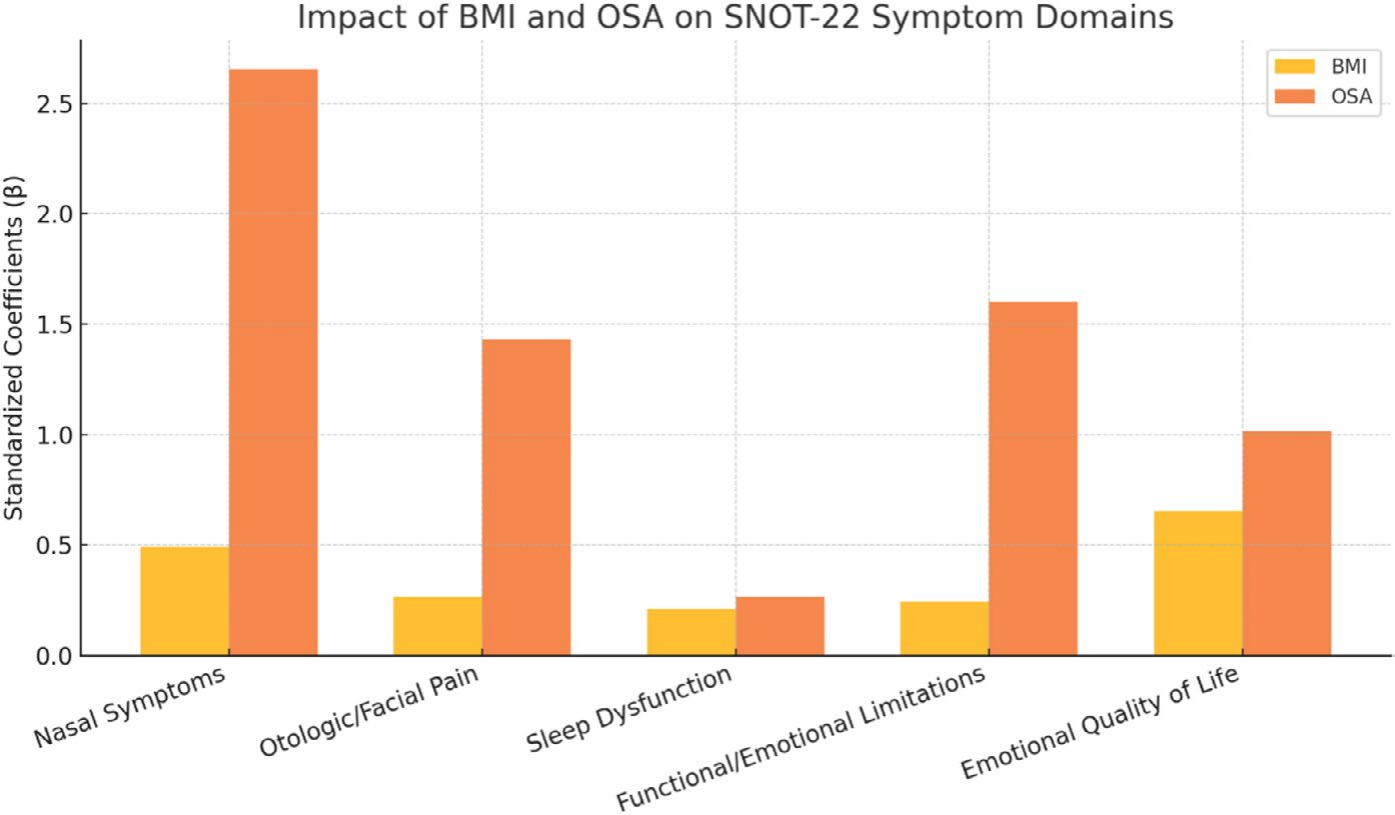
Standardised effects of BMI and sleep apnoea on SNOT‐22 symptom domains.

These findings suggest that BMI exerts a broad impact across all symptom domains in CRSwNP, while OSA may selectively influence domains more directly related to upper airway physiology and daily functioning as illustrated in Figure 2.

These associations are detailed in Table 4, which presents the standardised regression coefficients (*β*), corresponding *p*‐values and adjusted *R* ^2^ values for each of the five SNOT‐22 symptom domains. The results confirm that BMI significantly influences all domains, with the strongest effect observed in emotional QoL. In contrast, OSA shows selective significance, particularly in nasal, otologic/facial and functional/emotional domains.

**TABLE 4:** Multivariate regression analysis of BMI and OSA on SNOT‐22 symptom domains in CRSwNP.

SNOT‐22 domain	Adjusted *R* ^2^	*β* (BMI)	*p* (BMI)	*β* (OSA)	*p* (OSA)
Nasal symptoms	0.234	0.491	< 0.001	2.655	< 0.001
Otologic/facial pain	0.194	0.263	< 0.001	1.433	< 0.001
Sleep dysfunction	0.053	0.21	< 0.001	0.263	0.635
Functional/emotional limitations	0.127	0.243	< 0.001	1.6	0.005
Emotional quality of life	0.111	0.652	< 0.001	1.016	0.407

*Note*: Multivariate linear regression models were applied for each SNOT‐22 domain using BMI (continuous) and OSA (binary) as predictors. Statistically significant effects (*p* < 0.05) are highlighted.

## 4 Discussion

This study investigated sociodemographic and clinical variables associated with disease severity in patients with chronic rhinosinusitis with nasal polyps (CRSwNP). The results identify BMI and sleep apnoea as independent predictors of more severe nasal symptoms and worse disease‐specific QoL, as assessed by NOSE‐E and SNOT‐22 scores. Although smoking showed an association in the bivariate analysis, it did not retain significance in the multivariate models. These findings support the concept of CRSwNP as a systemic inflammatory condition influenced by modifiable comorbidities.

Several mechanisms may underlie these associations. Obesity is a recognised low‐grade systemic inflammatory state, characterised by elevated levels of adipokines and cytokines such as IL‐6 and TNF‐α, which can amplify sinonasal inflammation and contribute to mucosal oedema and polyp growth [14]. Furthermore, obesity is associated with impaired mucociliary clearance and altered nasal airflow dynamics, potentially worsening obstruction symptoms [15].

The relationship between OSA and CRSwNP has been highlighted in recent literature [7, 10]. Repetitive episodes of intermittent hypoxia in OSA are known to activate inflammatory pathways and oxidative stress, which may further impair sinonasal mucosal integrity and clearance mechanisms. Moreover, the pathophysiological overlap between both conditions may involve shared mechanisms such as type 2 cytokine overexpression, epithelial barrier dysfunction, mucosal oedema and the effects of intermittent nocturnal hypoxia, which could exacerbate upper airway inflammation and symptom burden in CRSwNP. Additionally, poor sleep quality may exacerbate symptom perception and reduce patients' tolerance to sinonasal discomfort. These findings emphasise the importance of identifying and managing OSA in CRSwNP, as interventions such as CPAP therapy may improve both sleep‐related and sinonasal symptoms [16].

The explanatory power of our multivariate models was moderate (adjusted *R* ^2^ = 0.224 for NOSE‐E and 0.242 for SNOT‐22), indicating that other relevant predictors remain unaccounted for. Future studies should consider including objective inflammatory markers (e.g., blood eosinophils, periostin, IL‐5), microbiome data, adherence to medical treatments and social determinants of health [17, 18]. Incorporating these variables could refine predictive models and support more personalised treatment strategies.

Moreover, the heterogeneity of our sample—with patients ranging from newly diagnosed individuals to those receiving corticosteroids, biologic agents, or having undergone prior ESS—represents a strength of the study. This diversity mirrors real‐world clinical practice and enhances the external validity of our findings, allowing for broader applicability across different treatment contexts.

The subdomain analysis of the SNOT‐22 revealed that BMI was consistently associated with a higher symptom burden across all domains, suggesting a broad systemic influence of obesity on sinonasal health and overall QoL in patients with CRSwNP. In contrast, the presence of OSA showed a more selective pattern, with significant associations in the nasal, otologic/facial pain and functional/emotional domains, but not in the sleep or emotional quality subdomains. This finding may reflect an overlap between upper airway obstruction and local nasal symptoms, as well as the indirect effect of OSA on daily functioning, without necessarily influencing sleep quality as captured by patient perception. Interestingly, OSA was not significantly associated with the sleep dysfunction subdomain of the SNOT‐22. This may be due to the fact that many patients with diagnosed OSA were already under treatment with CPAP or other supportive measures, potentially mitigating the perceived impact on sleep‐related symptoms. Moreover, the sleep items in CRSwNP instruments predominantly capture nocturnal symptoms driven by sinonasal inflammation (e.g., congestion‐related awakenings and daytime tiredness), which may not align with OSA‐specific physiological disturbance; thus, variance in this subdomain may be dominated by inflammatory nasal burden rather than BMI/OSA per se. Finally, other factors such as chronic inflammation, fatigue due to systemic disease, or medication effects may confound the relationship between OSA and subjective sleep quality in this population. These results emphasise the clinical relevance of stratifying symptom burden by domain when evaluating the impact of systemic comorbidities in CRSwNP. Subdomain‐level analysis may aid clinicians in tailoring interventions to the symptom profile most affected by each comorbidity.

In recent years, type 2 inflammatory markers have become central to guiding biologic therapy for CRSwNP. Agents such as dupilumab, omalizumab and mepolizumab have demonstrated significant improvements in symptom control and QoL [4, 19]. While eligibility is often based on eosinophilia, comorbid asthma and polyp burden, clinical predictors such as BMI and OSA may also help identify patients with more refractory disease and greater potential benefit from biologics. Recent data suggest that higher systemic inflammatory burden may be associated with improved response to dupilumab [20].

Interestingly, smoking was not retained as a significant predictor in our final models, despite previous associations with impaired mucociliary function and poor postoperative outcomes [21, 22]. This finding may reflect a relatively lower impact of tobacco exposure in the presence of more dominant systemic factors such as obesity and OSA or variability in smoking intensity and duration across individuals.

A notable strength of our study is the simultaneous use of two validated PROMs: SNOT‐22 and NOSE‐E. While the SNOT‐22 provides a broad overview of symptoms and their impact on daily life, the NOSE‐E focuses specifically on nasal obstruction, allowing for a more nuanced evaluation of disease burden [23]. This dual approach enhances the precision of symptom assessment and may improve clinical decision‐making.

However, the study has limitations. Its cross‐sectional design precludes causal inference. Biomarkers of inflammation and the lack of objective endoscopic or radiologic severity scores limit anatomical characterisation but are consistent with the study's focus on subjective symptom burden and QoL. Additionally, the study was conducted in a single centre and may not be generalisable to other populations. Larger, multicentre studies integrating biological, behavioural and environmental variables are needed to validate and extend these findings [17, 24]. Moreover, OSA status was clinically ascertained without systematic severity (e.g., apnoea–hypopnea index) or CPAP adherence data, which could bias domain‐specific estimates—particularly in the Sleep subdomain—towards the null.

In conclusion, this study identifies obesity and sleep apnoea as independent predictors of greater symptom severity and reduced QoL in patients with CRSwNP. These findings highlight the importance of integrating the assessment and management of systemic comorbidities into CRSwNP care pathways. Weight management and OSA screening should be considered essential components of comprehensive CRSwNP evaluation. Future research should evaluate whether targeted interventions in these areas can improve patient‐reported outcomes and response to advanced therapies, including biologics.

## Author Contributions

**Javier Modesto García‐Fernández:** principal investigator, conceptualisation, study design, methodology, formal analysis, writing – original draft, review and editing. **Miguel Ángel Feliz‐Fernández:** investigation, data collection, writing – review and editing. **María Soledad Sánchez‐Torices:** investigation, resources, writing – review and editing. **María Pilar Gómez‐Gallego:** investigation, resources, writing – review and editing. **Rafael Lomas‐Vega:** supervision, methodology, formal analysis, writing – review and editing. **María Alharilla Montilla‐Ibáñez:** investigation, data collection, project administration, formal analysis, supervision, writing – review and editing.

## Ethics Statement

Although the study did not undergo formal ethical committee review due to its retrospective and descriptive nature, all patients provided written informed consent for the publication of their clinical information and photographs.

## Conflicts of Interest

The authors declare no conflicts of interest.

## Data Availability

The data that support the findings of this study are available on request from the corresponding author. The data are not publicly available due to privacy or ethical restrictions.
